# Wilson’s Disease with Autoimmune Hepatitis Manifestation in a Pediatric Patient: A Case Report and Literature Review

**DOI:** 10.3390/jcm14248798

**Published:** 2025-12-12

**Authors:** Nicoleta Gimiga, Gabriela Păduraru, Laura-Iulia Bozomitu, Gabriela Ghiga, Monica Cristina Pânzaru, Lăcrămioara Ionela Butnariu, Ana Maria Scurtu, Elena Cojocaru, Vasile Valeriu Lupu, Laura-Mihaela Trandafir

**Affiliations:** 1 Department of Mother and Child, Faculty of Medicine, “Grigore T. Popa” University of Medicine and Pharmacy, 700115 Iasi, Romania; nicoleta.chiticariu@umfiasi.ro (N.G.); laura.bozomitu@umfiasi.ro (L.-I.B.); vasile.lupu@umfiasi.ro (V.V.L.); laura.trandafir@umfiasi.ro (L.-M.T.); 2 Department of Medical Genetics, Faculty of Medicine, “Grigore T. Popa” University of Medicine and Pharmacy, 700115 Iasi, Romania; monica.panzaru@umfiasi.ro (M.C.P.); ionela.butnariu@umfiasi.ro (L.I.B.); 3 Department of Surgery, Saint Mary’s Children Emergency Hospital, 700309 Iasi, Romania; a_m_scurtu@yahoo.com; 4 Department of Morphofunctional Science, “Grigore T. Popa” University of Medicine and Pharmacy, 700115 Iasi, Romania; elena2.cojocaru@umfiasi.ro

**Keywords:** Wilson’s disease, autoimmune hepatitis, child, overlap

## Abstract

**Introduction**: Wilson’s disease (WD) and autoimmune hepatitis (AIH) are important causes of acute and chronic hepatitis; each can lead to serious complications. The coexistence of these two diseases in the same patient is rare and poses significant diagnostic and therapeutic challenges. The pathophysiological mechanism in WD involves hepatocellular necrosis and the exposure of intracellular antigens to the immune system, resulting in the production of low-titer autoantibodies, which may be misleading and complicate the differentiation between WD and AIH. **Case Presentation**: We report the case of an 11-year-old girl admitted with abdominal pain, fatigue, and scleral jaundice. Physical examination revealed mild hepatomegaly without splenomegaly. Laboratory investigations were consistent with Wilson’s disease, and treatment with D-penicillamine was initiated. The initial clinical course was favorable; however, six weeks later, the patient again presented with acute hepatitis. A liver biopsy with histochemical analysis revealed findings highly suggestive of both Wilson’s disease and autoimmune hepatitis, confirming a dual diagnosis. **Conclusions**: The overlap of clinical and biochemical features between AIH and WD can delay accurate diagnosis and treatment, potentially affecting patient outcomes. Although the coexistence of Wilson’s disease and autoimmune hepatitis in the same child is rare, clinicians should maintain a high index of suspicion, given the complex management and the risk of complications associated with both disorders.

## 1. Introduction

Wilson’s disease (WD) is a rare autosomal recessive disorder resulting from mutations in the *ATP7B* gene, which is involved in copper transport. The ATP7B protein mediates biliary copper excretion, and its deficiency or reduced function leads to copper accumulation in the liver and other organs [1].

Establishing a WD diagnosis can be challenging because the clinical presentation in children is highly variable and specific laboratory markers are lacking [2]. Clinical manifestations of Wilson’s disease in children range from asymptomatic liver involvement to cirrhosis or acute liver failure. Moreover, at this age, neurological and psychiatric symptoms are rare.

Autoimmune hepatitis (AIH) is an inflammatory liver disease with a wide clinical spectrum, ranging from isolated acute or chronic hepatocellular injury to acute liver failure (ALF) [3].

AIH is characterized by the presence of autoantibodies, elevated immunoglobulin G (IgG) levels, and histological findings of lymphocytic or lymphoplasmacytic infiltration with interface hepatitis [4].

Diagnosing AIH is challenging because there is no single pathognomonic marker. AIH-associated autoantibodies may also appear in acute liver injury caused by other etiologies, and some liver diseases—such as chronic viral hepatitis—can also present with elevated serum globulin or IgG levels.

Wilson’s disease and autoimmune hepatitis are two conditions that can be difficult to differentiate, yet both may cause acute or chronic hepatitis and can lead to serious complications. The coexistence of both diseases in the same patient, particularly in children, is rare.

The pathophysiological mechanism in WD involves hepatocellular necrosis and the exposure of intracellular antigens to the immune system, resulting in the production of low-titer autoantibodies, which can be misleading when differentiating WD from AIH [5].

Distinguishing between these two entities is often challenging for pediatric gastroenterologists, meaning some cases of WD are initially misdiagnosed as AIH. In such patients, a partial response to corticosteroids and azathioprine may be observed. Therefore, screening for WD is recommended in patients initially diagnosed with AIH, especially when the response to immunosuppressive therapy is suboptimal. In such situations, combined treatment with corticosteroids and D-penicillamine may be highly effective [6].

Here, we present a case of acute hepatitis initially suggestive of WD, which subsequently evolved to exhibit overlapping features of both Wilson’s disease and autoimmune hepatitis.

## 2. Case Presentation

An 11-year-old girl was admitted to our hospital with a 7-day history of progressive abdominal pain, fatigue, and scleral jaundice.

In terms of her family history, the patient’s mother had autoimmune thyroiditis. Her past medical history was otherwise unremarkable, except for an episode of marked liver cytolysis (transaminase levels approximately ten times the ULN) one year earlier, at which time she was diagnosed with Epstein–Barr virus infection. No clinical or laboratory follow-up was performed after that episode.

On physical examination at admission, the patient was in poor general condition. She had mild hepatomegaly without splenomegaly, upper abdominal tenderness, pale skin, and scleral jaundice. Her vital signs were as follows: body temperature 36.7 °C, blood pressure 110/60 mmHg, heart rate 92 beats/min, and respiratory rate 19 breaths/min.

Initial laboratory investigations revealed mild anemia, direct hyperbilirubinemia, and severe hepatocellular cytolysis (approximately 15 times ULN). Coagulation tests were abnormal, showing prolonged prothrombin time and an increased international normalized ratio (INR). The laboratory findings are summarized in Table 1.

Because neither the personal nor family medical history suggested a specific diagnosis, we initiated a broad etiological workup including viral hepatitis, Wilson’s disease, and autoimmune hepatitis.

Serological tests for hepatitis A, B, and C viruses, Epstein–Barr virus, cytomegalovirus, and herpes simplex virus were all negative for IgM antibodies.

Additional investigations included serum ceruloplasmin, serum copper, 24 hours urine copper excretion, total cholesterol, triglycerides, immunoglobulin G (IgG), and autoantibody profiles (anti–liver-kidney microsome type 1 antibody, antinuclear antibody, anti–smooth muscle antibody, antimitochondrial antibody, and antiparietal cell antibody). All these parameters were within normal limits. The results are summarized in Table 2.

An abdominal ultrasound revealed a homogeneous liver echostructure without focal or space-occupying lesions; the liver span measured approximately 125 mm. The spleen had a normal echostructure and measured 110 mm.

An ophthalmologic examination using slit-lamp evaluation demonstrated the presence of a Kayser–Fleischer ring. Genetic testing for Wilson’s disease identified a homozygous ATP7B H1069Q mutation, confirming the diagnosis.

Our patient had a Leipzig score of 8 (a score >4 is considered positive for a WD diagnosis) [7,8].

Treatment with D-penicillamine was initiated, and the patient showed a favorable biochemical response. AST/ALT values after 2 weeks of d-penicillamine treatment were 180 UI/l and 197 UI/L, respectively.

The patient was subsequently discharged in good general condition, without scleral jaundice or abdominal pain, and was advised to continue regular follow-up. After 30 days of therapy, liver enzyme levels declined and approached normal values.

After six weeks, the patient was readmitted with recurrent fatigue, jaundice, and elevated liver enzymes (approximately ten times ULN).

Further evaluation for autoimmune hepatitis (AIH) was repeated and revealed elevated immunoglobulin G levels (1828 mg/dL). Tests for anti–liver-kidney microsomal type 1 antibody, antinuclear antibody, anti–smooth muscle antibody, and antimitochondrial antibody were negative.

Given the biochemical profile and clinical evolution, a liver biopsy was performed. Histopathological examination demonstrated remodeled hepatic architecture with regenerative nodules separated by fibrous septa. A diffuse and nodular lymphoplasmacytic inflammatory infiltrate was observed. Hepatocytes exhibited granular–vacuolar degeneration, mild nuclear enlargement, and occasional binucleation, accompanied by a mild interhepatocytic lymphoplasmacytic infiltrate, consistent with interface hepatitis, mild fibrosis, and bile duct proliferation.

**Figure 1:**
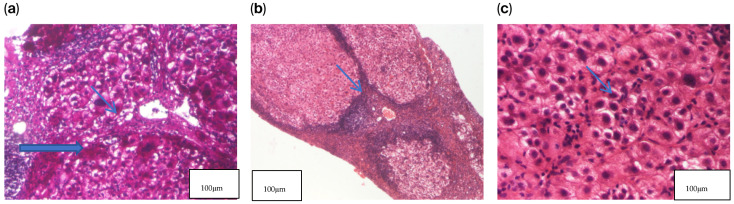
Liver biopsy—histopathological examination. (**a**) HE2 × 200 Hepatocytes exhibiting granular–vacuolar degeneration and mild nuclear enlargement, with occasional binucleated cells and a mild interhepatocytic lymphoplasmacytic infiltrate. (**b**) HE1 × 40 Hepatic parenchyma showing remodeled architecture with regenerative nodules separated by fibrous septa, associated with a diffuse and nodular lymphoplasmacytic inflammatory infiltrate. (**c**) PAS × 100 Regenerative nodules composed of hepatocytes with granular–vacuolar cytoplasm and lymphoplasmacytic inflammation within the portal–biliary spaces, accompanied by mild fibrosis and bile duct proliferation.

Histochemical staining with rhodanine and orcein was negative. Special stains for copper did not reveal copper-laden hepatocytes (Figure 1).

The simplified AIH score in our patient was 6 (2 points for an Ig G level above 1.1×ULN, 2 points for typical liver histology of AIH–interface hepatitis, and 2 points for exclusion of viral hepatitis). A score of 6 was considered sufficient for the diagnosis of AIH and for initiating treatment with oral prednisone at a dose of 1 mg/kg/day while D-penicillamine was continued. After two weeks, azathioprine was added, resulting in a marked clinical and biochemical improvement. Liver enzyme levels began to decline after three weeks of therapy, reaching near-normal values after two months of treatment with d-penicillamine, prednisone, and azathioprine (aspartate transaminase 35 U/L; alanine transaminase 38 U/L). Total and direct bilirubin levels normalized after two months (0.5 mg/dL and 0.1 mg/dL, respectively). Prothrombin time and international normalized ratio (INR) returned to normal ranges after three months of therapy.

## 3. Discussions

Establishing an accurate etiological diagnosis in cases of acute hepatitis represents a significant challenge in pediatric gastroenterology [7].

Several cases have been reported describing patients with classical manifestations of Wilson’s disease (WD) and concurrent features of autoimmune hepatitis (AIH).

It is often difficult to determine the correct diagnosis in such patients, especially when clinical and laboratory findings support both WD and AIH. Autoantibodies may be positive in WD owing to hepatocyte necrosis, particularly in the early stages of the disease, further complicating the differential diagnosis [8].

Liver biopsy can be helpful if histochemical stains for copper or copper-associated proteins, such as rhodamine, provide qualitative evidence of increased hepatic copper. However, despite elevated hepatic copper content, these stains are frequently negative in WD patients [9].

In our case, we discuss seronegative AIH because Wilson’s disease can itself suppress antibody production; copper accumulation leads to hepatocyte injury and immune exhaustion, lymphocyte dysfunction, and loss of synthetic function, including reduced globulin production. This can inhibit the formation of detectable autoantibodies, creating a false impression of seronegativity even when autoimmune injury is present. Additionally, copper toxicity can trigger immune activation, and cytokine dysregulation in WD may predispose to autoimmunity. The clinical course strongly suggests AIH, including marked inflammation disproportionate to copper burden, response to steroids (rapid improvement), and the persistence of hepatitis despite copper chelation.

The 24 hour urine copper excretion test remains one of the most reliable diagnostic tools for WD. However, in asymptomatic children or those with mild liver disease, urinary copper levels may be within normal limits. The reported optimal diagnostic cutoff for basal urinary copper excretion is 40 mg/24 h (0.65 mmol/24 h), with a sensitivity of 78.9% and a specificity of 87.9% [10].

The penicillamine challenge test (0.5 g of D-penicillamine administered at the beginning of urine collection and again 12 h later) is considered unreliable for excluding WD in asymptomatic children, demonstrating a low sensitivity (12–46%) at the diagnostic cutoff of 1575 mg/24 h (25 mmol/24 h), as was the case in our patient [11].

Wilson’s disease is caused by pathogenic variants in ATP7B, a gene that is primarily expressed in the liver, brain, kidney, and placenta.

The ATP7B gene consists of 21 exons located on chromosome 13q14.3. More than 700 variants and polymorphisms have been described in WD patients, of which over 300 are considered likely pathogenic [12,13]. Missense and nonsense mutations, deletions, and insertions have been reported across nearly all 21 exons. Most patients are compound heterozygotes, which contributes to the wide variability of clinical phenotypes. ATP7B mutations also exhibit marked geographical variability [14].

In our patient, genetic testing for Wilson’s disease identified a homozygous ATP7B H1069Q mutation, confirming the diagnosis.

Liver biopsy remains an important diagnostic and management tool in patients with autoimmune liver disease.

However, it cannot be used as a standalone diagnostic test, and interpretation requires correlation with clinical, biochemical, and immunological data [15].

Liver biopsy also provides information on disease severity, including inflammatory activity and fibrosis stage, which have implications for prognosis and therapy.

Histological findings can either support a diagnosis of AIH or exclude other hepatic disorders such as fatty liver disease or chronic biliary pathology.

The classical histological feature of chronic autoimmune hepatitis is a plasma-cell–rich mononuclear infiltrate, predominantly involving the portal and periportal regions [16].

However, the absence of abundant plasma cells does not exclude AIH, as mild plasma cell infiltrates may also occur in other chronic liver diseases associated with portal inflammation. Interface hepatitis is considered a key histopathological feature of AIH [17].

There is ongoing controversy regarding the role of liver biopsy. While international guidelines continue to recommend routine biopsy in suspected cases of AIH, some experts suggest restricting biopsy to equivocal or atypical presentations, such as when autoantibody titers are low or absent or when alternative diagnoses (e.g., fatty liver disease, biliary disease) are suspected [18].

Approximately 10–20% of patients with biochemical and histological features suggestive of AIH lack detectable conventional autoantibodies [19].

Many of these cases exhibit elevated serum IgG levels and respond favorably to corticosteroid therapy, suggesting an autoantibody-negative variant of AIH.

This hypothesis is supported by evidence that some of these patients later seroconvert and become autoantibody-positive during follow-up [20].

When patients with a presumed diagnosis of AIH show a poor response to corticosteroids, screening for WD is recommended [21].

Although this case initially appeared to represent acute hepatitis secondary to Wilson’s disease, the unfavorable biochemical response to D-penicillamine therapy necessitated a more comprehensive evaluation.

Several cases of WD with superimposed autoimmune manifestations have been described, and in such instances, combined therapy with D-penicillamine and corticosteroids may be beneficial, as observed in our patient [22].

The coexistence of WD and AIH is not unique among dual liver pathologies. Other combinations have been reported, such as AIH with non-alcoholic fatty liver disease (NAFLD) [23].

Corticosteroid therapy, the mainstay of AIH treatment, may induce insulin resistance, obesity, and hepatic steatosis, thereby predisposing patients to NAFLD.

Given the rising incidence of obesity and NAFLD in both adults and children, it is essential to evaluate for AIH–NAFLD overlap prior to initiating steroid therapy, and liver biopsy remains the gold standard in these cases.

Dara et al. reported a similar case of a 10-year-old boy presenting with overlapping features of WD and AIH, who responded well to prednisolone, azathioprine, and D-penicillaminetherapy [24].

Mazumder et al. described a case of an 8-year-old girl initially diagnosed with WD; subsequent investigations confirmed coexisting AIH, and combined therapy was well tolerated with a favorable outcome [25].

Bekyarova et al. reported a 15-year-old boy diagnosed with AIH who received corticosteroids and was discharged, but was readmitted three days later in critical condition. Despite intensive resuscitation, the patient died. Autopsy findings revealed mixed micronodular and macronodular cirrhosis, hepatosplenomegaly, ascites, icterus, telangiectasias, subcutaneous hemorrhages, hypogonadism, and chronic calculous cholecystitis consistent with untreated WD [26].

D-penicillamine may induce autoimmune hepatitis and could be a plausible complementary explanation for the patient’s biochemical and immunological findings. Considering the timeline of symptom onset and the immunomodulatory properties of D-penicillamine, this possibility merits acknowledgment in the context of the disease course. Distinguishing between true autoimmune hepatitis and drug-induced immune-mediated liver injury can be challenging, particularly when both conditions may coexist or overlap in Wilson’s disease. We also highlight that the improvement observed after treatment adjustment further supports the importance of considering this mechanism in clinical practice.

This tragic case underscores the importance of considering WD in the differential diagnosis of acute or chronic hepatitis.

In the context of an 11-year-old girl presenting with both WD and AIH, these observations raise the possibility that copper-induced immune activation could play a role in triggering or exacerbating autoimmune liver injury. Such overlap may complicate the clinical picture, affecting both diagnostic interpretation and therapeutic decision-making. Nevertheless, the immunological dimension—including cytokine alterations, autoantibody patterns, and potential genetic susceptibility—was not explored in the current report, leaving open important questions about the underlying pathophysiological link between these two conditions.

## 4. Conclusions

There have been reported cases of patients with classical manifestations of Wilson’s disease (WD) who also exhibit features of autoimmune hepatitis (AIH).

Our patient initially presented with typical features of Wilson’s disease and was later found to have coexisting autoimmune hepatitis.

Differentiating between these two entities is crucial, as autoantibodies may be positive in the early stages of WD, potentially leading to diagnostic confusion. Clinicians should therefore consider the possibility of concurrent WD and AIH in patients where establishing a definitive diagnosis is challenging.

The coexistence of Wilson’s disease and autoimmune hepatitis in the same patient is rare, but this possibility warrants a high level of clinical awareness due to the complexity of management and potential complications associated with these disorders.

## Figures and Tables

**Table 1:** Primary Laboratory Investigation.

Marker	Normal Value	Value	Marker	Normal Value	Value	Marker	Normal Value	Value
WBC	4–9 × 10^3^ /μL	5.6	AST	17–40 U/L	**454**	Urea	20–40 mg/dL	24
RBC	4.1–5.1 × 10^6^ /μL	3.2	ALT	18–51 U/L	**511**	Cr	0.3–0.6 mg/dL	0.5
Hb	11.5–15 g/dL	**9.4**	Uric acid	2–5.4 mg/dL	2.1	Na	135–145 meq/L	136
Platelet	150–400 × 10^3^/μL	189	Bilirubin (total, direct)	0.1–0.7 mg/dL 0.1–0.2 mg/dL	**2.7** **2.1**	K	4.1–4.9 meq/L	4.2
Reticulocytes	2–6%	2.7%	Alkaline phosphatase	32–300 U/L	378	Ca	8.8–10.4 mg/dL	8.9
MCV	80–100 fL	92.7	Glucose	60–90 mg/dL	72	Phosphate	4.5–7 mg/dL	4.3
Coombs direct	negative	negative	PT, INR	11–14 s 0.8–1.2	**19.2** **1.7**	Total protein	6–8.3 g/dL	**8.2**
ESR	˂12 mm/h	**34**	PTT	25–32 s	**42 s**	Albumin	3.5–5.2 g/dL	**3.2**

**Table 2:** Specific Laboratory Investigation.

Marker	Normal Value	Value	Marker	Normal Value	Value
Immunoglobulin G	600–1300 mg/dL	1273	Ceruloplasmin	20.5–43 mg/dL	**11**
AMA	˂1/20	1/20	Serum copper	67–128 μg/dL	**141**
ANA	˂1/80	1/10	ASMA	˂1/32	˂1/32
HBs Ag	˂0.9	0.2	Anti-LKM1	˂1/40	1/20
HAV Ab (IgM)	˂1 U/L	0.3	Alpha 1 antitrypsin	0.9–2 g/L	1.3
HCV-Ab Ig M	˂1 UI/L	0.09	24 h urine copper (1 day after challenge with 2 × 0.5 g D-penicillamine	˂60 μg/24 h	**980**
Total cholesterol	50–170 mg/dL	98	Triglycerides	44–150 mg/dL	103

## Data Availability

Data are contained within the article.

## Abbreviations

The following abbreviations are used in this manuscript:

ALT	alanine transaminase
AST	aspartate aminotransferase
Ca	Calcium
Cr	Creatinine
ESR	erythrocyte sedimentation rate
Hb	Hemoglobin
INR	international normalized ration
K	potassium
MCV	mean cell volume
Na	Sodium
Pt	prothrombin time
PTT	partial thromboplastin time
RBC	red blood cell
WBC	white blood cel
ANA	anti-nuclear antibody
AMA	anti-mitochondrial antibody
ASMA	anti-smooth muscle antibody
Anti-LKM1	anti-liver kidney microsome type 1 antibody
HAV Ab	hepatitis A virus antibody
HBs Ag	hepatitis B surface antigen
HCV-Ab	hepatitis C virus antibody
Ig G	immunoglobulin G
